# A Case of Pediatric Sjögren's Syndrome Diagnosed With Microcalcifications in Both Parotid Glands

**DOI:** 10.7759/cureus.72730

**Published:** 2024-10-30

**Authors:** Chihiro Sugiyama, Yuichi Takahashi, Tatsuo Fuchigami, Satoshi Sato, Ichiro Morioka

**Affiliations:** 1 Department of Pediatrics, IMS Fujimi General Hospital, Fujimi, JPN; 2 Department of Infection, Immunology and Allergy, Saitama Children's Medical Centre, Saitama, JPN; 3 Department of Pediatrics and Child Health, Nihon University School of Medicine, Tokyo, JPN

**Keywords:** children, chronic headache, malaise, parotid gland calcifications, sjögren's syndrome

## Abstract

Sjögren's syndrome (SS), an autoimmune disease primarily affecting the exocrine glands, particularly the lacrimal and salivary glands, typically manifests as sicca symptoms (dry mouth and dry eyes). However, these symptoms are uncommon in pediatric patients. We present a case of a 13-year-old boy diagnosed with SS following the detection of bilateral parotid gland microcalcifications during a computed tomography scan for headache evaluation. The patient presented with a chief complaint of persistent headaches and malaise. However, he did not have sicca symptoms. A plain head computed tomography scan revealed multiple microcalcifications within the bilateral parotid glands. The fat-suppressed T2-weighted magnetic resonance imaging scan showed bilateral parotid gland enlargement and scattered areas of high intensity within the parotid glands. The patient was diagnosed with SS based on a serological examination and salivary and lacrimal gland disorders. Treatment with hydroxychloroquine improved headache and fatigue substantially. The patient can now participate in sports and daily life activities without difficulty. These findings stress the importance of considering SS in the differential diagnosis of children with chronic headaches and malaise. The presence of calcification within the parotid gland should raise suspicion for SS, and imaging studies can be a valuable tool for diagnosis, providing crucial information for informed decision-making.

## Introduction

Sjögren's syndrome (SS) is an autoimmune disease characterized by inflammation of the exocrine glands, such as the lacrimal and salivary glands, resulting in sicca symptoms (dry mouth and dry eyes). SS is prevalent in middle-aged women but rare in children [1]. However, most pediatric patients with SS present with extra-glandular manifestations and do not exhibit the symptoms of sicca [2, 3]. Although the exocrine glands are damaged in patients with childhood SS, the decline in their function is mild, which complicates diagnosis [1].

Recently, parotid gland cysts and calcifications have been identified as new signs of SS in adult patients [4-6]. However, the lack of detailed analytical data and scarcity of reports on pediatric cases necessitate further research. Employing head computed tomography (CT) or magnetic resonance imaging (MRI), followed by antibody titer measurements and tests for exocrine gland disorders, can significantly help in the early diagnosis of childhood SS. Although cases of SS in children are sporadic, we present a unique pediatric case of microcalcifications of the bilateral parotid glands identified on CT during an examination of headache, which led to the diagnosis of SS.

## Case presentation

A 13-year-old boy presented with a chief complaint of persistent headaches, a condition that had been affecting his daily life since approximately age 11. Despite regular visits to his family doctor and a brain CT scan that showed no abnormalities, his condition deteriorated. Seven days before admission, his symptoms worsened, and three days later he visited the hospital owing to migrating systemic pain.

His medical history revealed allergic rhinitis and allergies to ticks, cedar, cypress, prawns, crabs, and squid. When he was six years old, he was hospitalized for nine days for pharyngotonsillitis and cervical lymphadenitis with high serum C-reactive protein levels and recurrent fever. He was treated with meropenem hydrate. The left parotid gland was mildly enlarged but internally regular on cervical CT.

At the age of seven, he was hospitalized for four days with parotitis and treated with only fluid replacement. He did not undergo imaging studies, including CT. The skin on his palms started peeling off around age nine, for which he received treatment from a dermatologist. When he was 13 years old, the patient contracted coronavirus disease 2019 (COVID-19).

His older brother is under care at the pediatric mental and neurological outpatient department for orthostatic dysregulation (OD) and social anxiety disorder.

At the time of admission to our hospital, the patient was 147 cm (-1.5 standard deviation (SD)) in height and weighed 48 kg (-0.2 SD). His body temperature was 37.0 °C, heart rate was 70 beats/min, and blood pressure was 110/57 mmHg. His general condition was stable. Neurological findings revealed no abnormalities. The parotid gland was not palpable. Thyroid gland enlargement was not observed. Examination of the chest and abdomen revealed no abnormal findings. No abnormalities in vitals or physical examination and no skin rash were observed.

Laboratory blood tests (Table 1) revealed mildly elevated total protein (8.8 g/dL) and amylase levels (141 U/L).

**Table 1:** Laboratory data ANA, anti-nuclear antibody; anti-ds-DNA Ab, anti-ds-DNA antibody; Ds-DNA, anti-double-stranded deoxyribonucleic acid antibody; SS-A, anti-SS-A/Ro antibody; SS-B, anti-SS-B/La antibody; BUN, blood urea nitrogen; C3, complement component 3; C4, complement component 4; CH50, 50% hemolytic complement activity; CRP, C-reactive protein; LDH, lactate dehydrogenase; IgG, immunoglobulin G; IgA, immunoglobulin A; IgM, immunoglobulin M; RF, rheumatoid factor; RBC, red blood cell; WBC, white blood cell

Test	Result	Reference Range		Units
WBC	4,500	(3,300–8,600)		µL
RBC hemoglobin	513 14.1	(435–555) (13.7–16.8)		×10^4^/µL g/dL
Platelet	174	(158–348)	×10^3^/µL	
BUN	11.8	(8–23)		mg/dL
Creatinine	0.45	(0.61–1.08)		mg/dL
CRP	0.09	(0.04–0.14)		mg/dL
Total protein	8.8	(6.6–8.1)		g/dL
Albumin	4.9	(4.1–5.1)		g/dL
LDH (IFCC)	169	(124–222)		U/L
Amylase (IFC)	141	(44–132)		U/L
IgG	2119	(870–1700)		mg/dL
IgA	301	(110–410)		mg/dL
IgM	125	(33–190)		mg/dL
C3	137.8	(86–160)		mg/dL
C4	23.6	(17.0–45.0)		mg/dL
CH50	44.2	(25.0–48.0)		CH50/mL
RF	59.2	(0–15)		IU/mL
ANA	1:40	(0–40)		
Ds-DNA	0.8	(0.0–10.0)		IU/mL
SS-A	>240.0	(0–10)		U/mL
SS-B	31.1	(0–10)		U/mL

**Figure 1:**
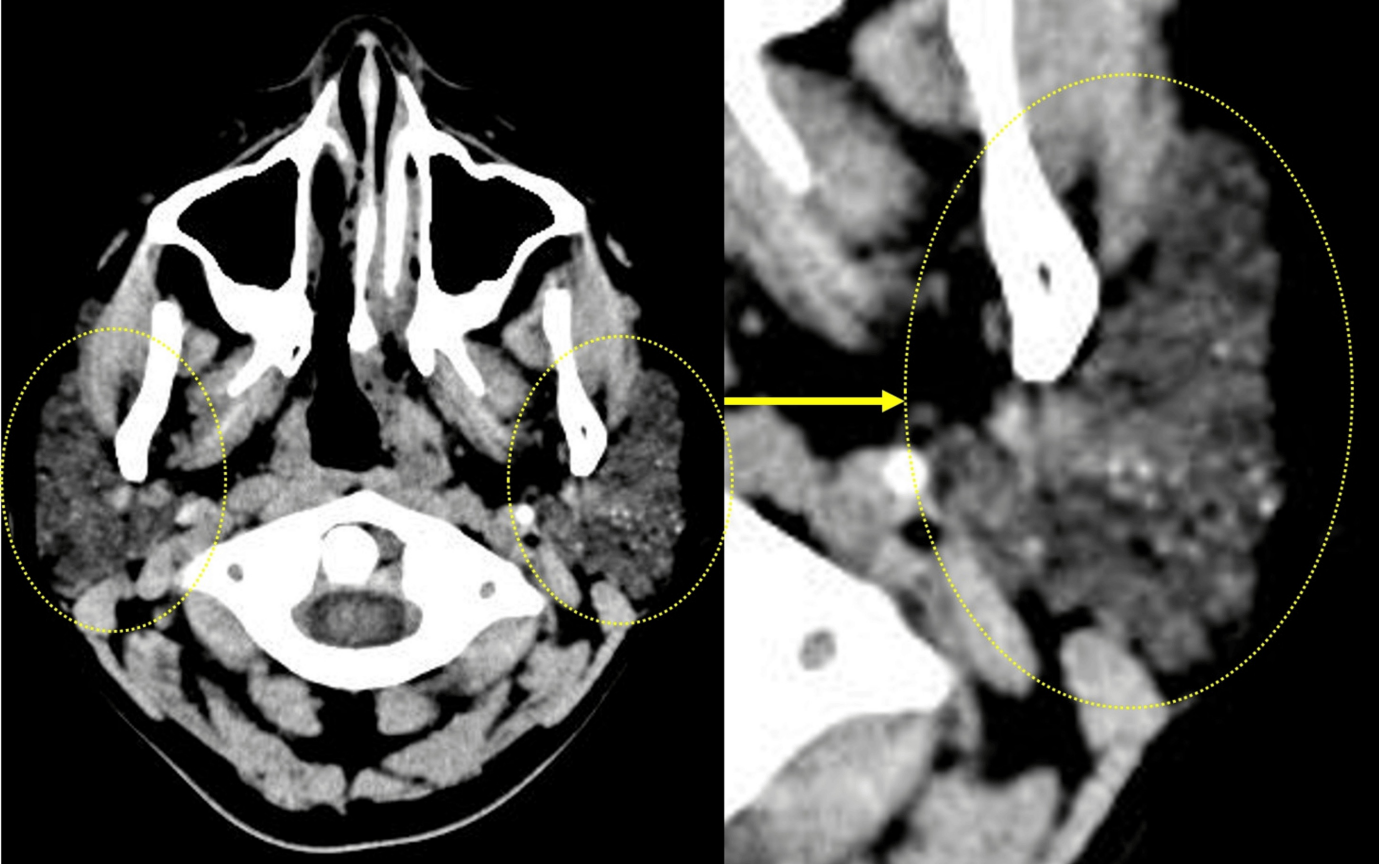
Computed tomography (CT) scan of the bilateral parotid glands A plain head CT scan shows multiple microcalcifications within the bilateral parotid glands, predominantly on the left side.

We used a comprehensive approach to examine headaches and fatigue, including a head CT scan and OD test. A plain head CT scan showed multiple microcalcifications within the bilateral parotid glands, predominantly on the left side (Figure 1). However, the CT scan performed two years prior for a headache examination revealed no prominent abnormal findings. Among the physical symptoms of OD, listed as differentials for complaints, 7/11 were applicable [7]. However, a new standing test did not indicate a decrease in blood pressure or an increase in pulse rate; therefore, the patient did not meet the diagnostic criteria for OD [7]. This thorough evaluation allowed us to rule out various conditions and focus on the correct diagnosis.

**Figure 2:**
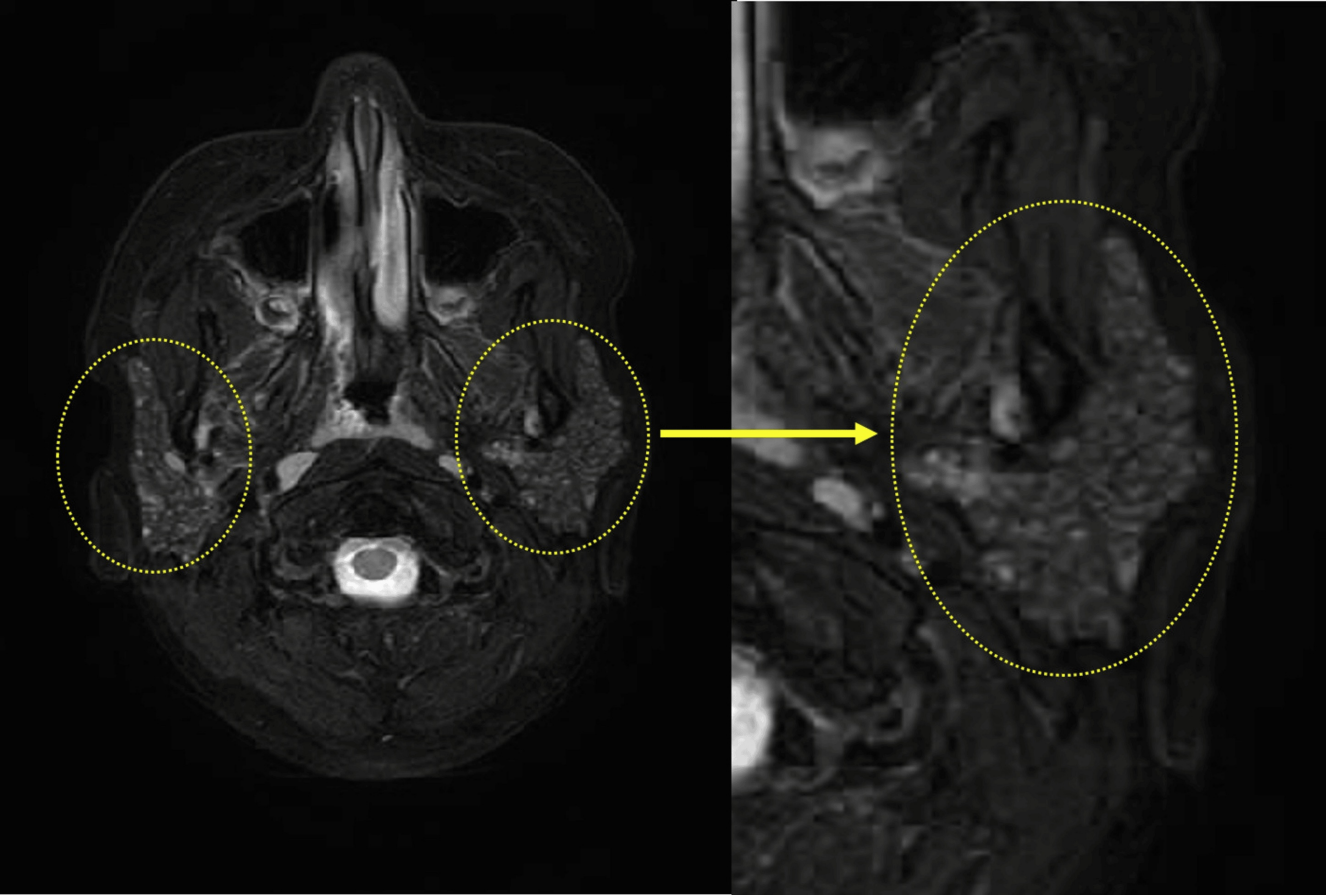
Magnetic resonance imaging (MRI) scan of the bilateral parotid glands The fat-suppressed T2-weighted MRI scan shows bilateral parotid gland enlargement and scattered findings of hyperintense areas within the parotid glands.

Fat-suppressed T2-weighted MRI scan revealed bilateral parotid gland enlargement and scattered areas of high intensity within the parotid glands (Figure 2).

We suspected a diagnosis of SS and performed a blood examination. The blood test results indicated elevated IgG (2119 mg/dL), C3 (137.8 mg/dL), rheumatoid factor (59.2 IU/mL), anti-SS-A antibody (>240.0 U/mL), and anti-SS-B antibody (31.1 U/mL).

Salivary gland scintigraphy was used to evaluate saliva uptake and secretion functions on the exocrine gland. Mildly decreased uptake in the parotid gland and decreased uptake and secretion in the submandibular gland were observed. These findings indicated that the major salivary glands were dysfunctional. The Schirmer test, used to assess lacrimal fluid retention, was negative, indicating sufficient tear production.

According to the diagnostic criteria outlined in the Japanese clinical practice guidance for SS in pediatric patients (2018), the serological examination score of 11 and salivary gland score of 1 [1] suggested a probable diagnosis of SS (serological score > 4 and salivary gland score = 1). Treatment with hydroxychloroquine (Plaquenil) at 200 mg/day was initiated, resulting in substantial improvement in his headache and fatigue symptoms. The positive response to treatment supported the effectiveness of our approach, and the patient can now participate in sports and daily life activities without difficulty.

## Discussion

SS is the fourth most common pediatric collagen disorder with a higher prevalence in girls than in boys [8]. Sicca symptoms are less common in children than in adults, with malaise and parotid swelling being more frequently reported [1, 3].

In clinical practice, many patients present with malaise as the primary complaint. The diagnostic guide states indefinite complaints as initial symptoms of SS, such as malaise, headache, mild fever, musculoskeletal pain, chest pain, nausea, abdominal pain, and dizziness [1]. Approximately half of the patients with chronic indefinite complaints test positive for antinuclear antibodies. Additionally, 10% of these patients test positive for anti-SS-A/Ro antibodies [9].

Given the challenges that children face in recognizing sicca symptoms, conducting medical interviews using specific questions is crucial for diagnosing pediatric SS. These questions, which include whether the patient needs to drink water when eating dry food, bad breath, parotid swelling or pain, dental caries, oral abnormalities, taste changes, and dry eye symptoms, are not just a formality but a key part of the diagnostic process.

Pediatric patients with SS present with a broad spectrum of clinical features, a low prevalence of sicca symptoms, and various clinical manifestations with multisystem involvement. The American-European Consensus Group [10] and American College of Rheumatology/European League Against Rheumatism [11] criteria have been established to diagnose SS. However, these criteria are not applicable to pediatric SS [12]. Hou et al. reported that the Japanese version of the Clinical Practice Guidance for SS in Pediatric Patients (2018) helped diagnose pediatric SS [13]. Accordingly, we used this version for diagnosis in this study.

Previous imaging studies of SS in adult patients reported the presence of multiple microlithiases in the bilateral parotid glands [4-6,14]. However, studies using CT and MRI to evaluate pediatric patients with SS are rare, and case reports of pediatric SS diagnosed with microcalcifications in both parotid glands are sporadic. This rarity underscores the uniqueness and significance of the present study. Takagi et al. [15] described the case of a 10-year-old girl with primary juvenile SS, noting changes in imaging findings over a seven-year period. The patient exhibited hyperintense spots suggestive of punctate sialectasis in both parotid glands on fat-suppressed T2-weighted MR imaging from 10 years of age, even before sicca symptoms developed. The patient in this study showed similar CT and MRI findings, without sicca symptoms.

Regarding the changes observed on imaging over time, the salivary glands initially appeared diffusely enlarged in the early stages and began to show calcification on CT in the chronic stage. However, the mechanisms underlying stone formation in patients with SS remain unclear. Reduced salivary flow and a high concentration of calcium salts in the saliva may be etiologic factors [16].

The MRI findings in a study of 90 patients, including children and adults, suggested that scattered high-intensity areas in the parotid gland are characteristic of SS in children [17], consistent with the findings of this study. The fat-suppressed T2-weighted MRI in this study demonstrated multiple high-intensity spots in the parotid gland, which also indicated lymphocyte infiltration in the glandular parenchyma and punctate sialectasis. This result supported the findings by Takagi et al [17].

## Conclusions

We reported a case of pediatric SS diagnosed with microcalcifications in both parotid glands. In pediatric SS, few patients complain of sicca symptoms, a characteristic of adult SS, and delayed diagnosis is problematic. However, early diagnosis and treatment can significantly improve the patient's quality of life and prevent organ dysfunctions during disease progression. Our findings underscore the importance of imaging studies in the diagnosis of pediatric SS. Children with chronic headaches and malaise should be included in the differential diagnosis of SS. Microcalcifications within the parotid gland can be a strong indicator of SS and imaging studies are invaluable tools for establishing a diagnosis.
